# Clinicopathologic features of remnant gastric cancer after curative distal gastrectomy according to previous reconstruction method: a retrospective cohort study

**DOI:** 10.1186/s12957-019-1740-3

**Published:** 2019-11-30

**Authors:** Yong-Eun Park, Sang-Woon Kim

**Affiliations:** Department of surgery, Yeungnam University Medical Center, Daegu, Korea

**Keywords:** Remnant gastric cancer, Reconstruction, Recurrence interval

## Abstract

**Background:**

Survival rate of patients treated for gastric cancer has increased due to early detection and improvements of surgical technique and chemotherapy. Increase in survival rate has led to an increase in the risk for remnant gastric cancer (RGC). The purpose of this study was to investigate clinicopathologic features of RGC according to previous reconstruction method and factors affecting the interval from previous curative distal gastrectomy for gastric cancer to RGC occurrence.

**Methods:**

Medical records of patients diagnosed with RGC at Yeungnam University Medical Center from January 2000 to December 2017 who had a history of distal gastrectomy with D2 LN dissection due to gastric cancer were reviewed retrospectively.

**Results:**

Forty-eight patients were enrolled in this study. The mean interval of 48 RGC patients was 105.6 months (8.8 years). RGC after Billroth II reconstruction recurred more often at anastomosis site than RGC after Billroth I reconstruction (*p* = 0.001). The mean interval of RGC after Billroth I reconstruction was 67 months, shorter than 119 months of RGC after Billroth II reconstruction (*p* = 0.003). On the contrary, interval showed no difference according to stage of previous gastric cancer, remnant gastric cancer, or sex (*p* = 0.810, 0.145, and 0.372, respectively).

**Conclusions:**

RGC after Billroth I reconstruction tends to arise earlier at non-anastomosis site than RGC after Billroth II. Therefore, we should examine non-anastomosis site carefully from the beginning of surveillance after gastric cancer surgery with Billroth I reconstruction for better outcome.

## Introduction

Gastric cancer is the fifth most common cancer and the third cause of death due to cancer in the world [1]. It is especially common in the Republic of Korea and Japan compared with its population. It is also the most common cancer in Korean males [2].

Remnant gastric cancer (RGC) is a carcinoma that develops in the remnant stomach after gastrectomy. RGC was first reported by Balfour without an exact nomenclature in 1922 as gastric cancer developing in patients operated for gastric ulcer [3]. Since the report by Balfour, carcinoma arising from remnant stomach after gastrectomy due to gastric cancer was also included in RGC. Its nomenclature and definition was not unified for a long period. Researchers have used various terms such as stump gastric cancer, gastric remnant cancer, and carcinoma in remnant stomach with un-unified definition about the initial disease and duration from previous gastrectomy. Some researchers defined RGC as gastric cancer detected more than 5 years after gastric cancer surgery while other researchers defined it as gastric cancer detected more than 10 years after gastric cancer surgery [4, 5]. Currently, RGC is defined as carcinoma arising from remnant stomach regardless of the initial disease or duration from previous surgery [6].

Recently, RGC after benign disease (RGC-B) has not increased as peptic ulcer gastrectomy has become rare due to development of PPI [7]. On the other hand, RGC after gastric cancer (RGC-M) has increased due to increased survival rate of gastric cancer patients because of early detection and improvement in surgical technique and chemotherapy [8]. Therefore, it is necessary to study clinicopathologic features of RGC after curative distal gastrectomy and establish reasonable follow-up examination plan for early detection of RGC which is essential for good prognosis.

This study was designed to examine clinicopathologic features of RGC-M based on previous reconstruction method and investigate clinicopathologic features affecting the interval from previous curative distal gastrectomy for gastric cancer to RGC occurrence.

## Methods

### Patients and study design

Among 4284 patients who underwent gastrectomy at Yeungnam University Medical Center (YUMC) from January 01, 2000 to December 31, 2017, medical records of 66 gastric cancer patients who had a history of gastrectomy were reviewed retrospectively.

**Fig. 1:**
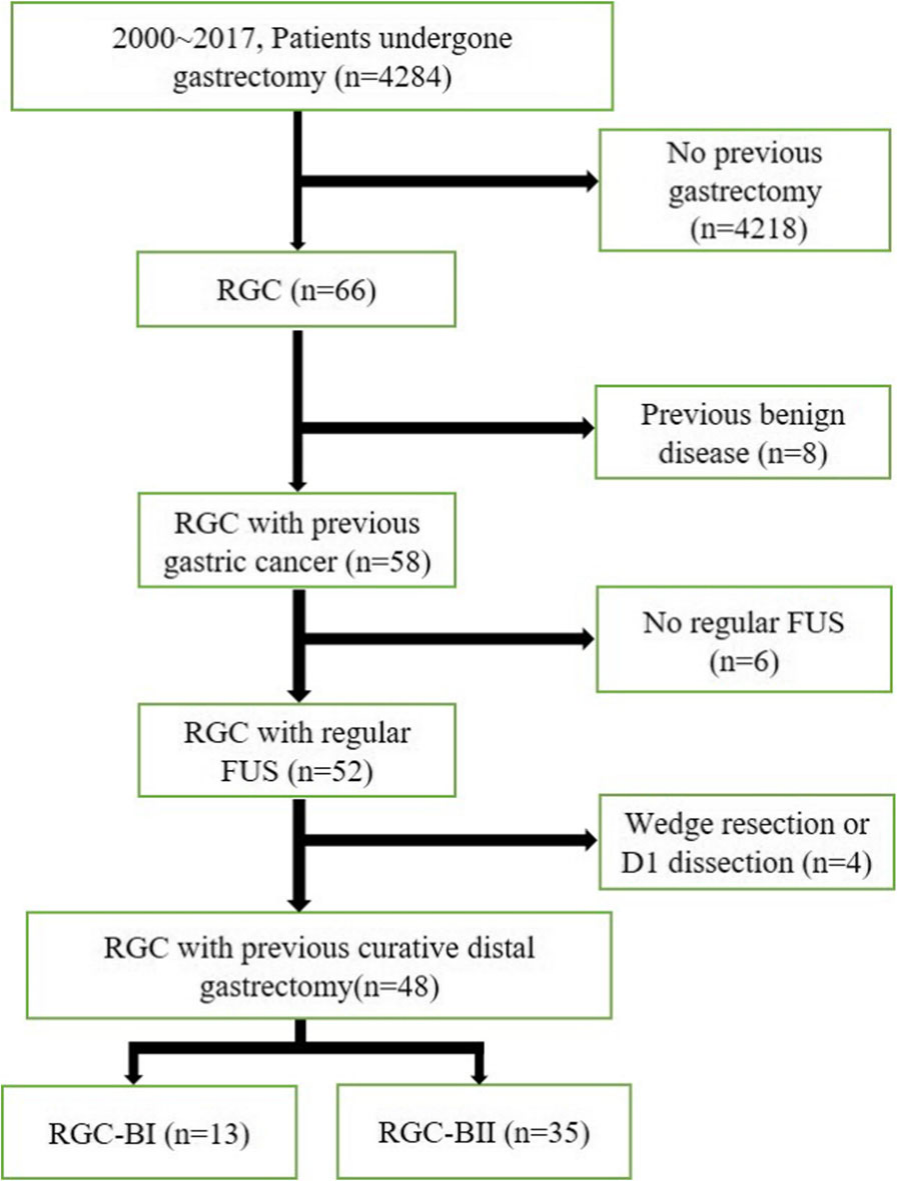
Patients selection flow chart. RGC, remnant gastric cancer; FUS, follow-up study; RGC-B I, RGC after Billroth I reconstruction; RGC-B II, RGC after Billroth II reconstruction

Patients who fulfilled the following criteria were eligible in this study: (1) previous gastrectomy for gastric cancer, (2) regular follow-up studies before diagnosis with RGC, and (3) D2 LN dissection with Billroth I or Billroth II reconstruction method. According to the eligible criteria, data of 48 patients were analyzed retrospectively after excluding 18 patients who failed to meet these criteria (Fig. 1).

### Decision of reconstruction method in distal gastrectomy

After D2 LN dissection was completed, great curvature on distal body of stomach was opened for visualization of the cancer lesion. The final decision on reconstruction method was done with reference to macroscopic findings and the location of the cancer to make tension-free anastomosis. Anastomosis between the duodenum or jejunum and the posterior wall of the remnant body of the stomach (close to greater curvature) was then performed.

### Operation method for RGC

Operation for 48 RGC patients was total gastrectomy with Roux-en-Y anastomosis. During RGC operation, residual peri-gastric LN dissection was done and LN 10 was removed in case of visible LN enlargement or RGC on greater curvature. If previous reconstruction method was Billroth II and infiltration of jejunum or LN metastasis was suspected, jejuno-mesenteric LN dissection was performed, including the origin of each involved jejunal artery. Splenectomy was performed if patients met the following criteria: (1) uncontrolled bleeding of spleen, (2) direct invasion of spleen, and (3) invasion of gastro-splenic ligament.

### Follow-up study after gastrectomy

For follow-up clinical studies, history taking, physical examination, serum tumor marker evaluation, simple chest X ray, esophagogastroduodenoscopy (EGD), and abdominal CT scan were carried out at intervals of 6 months for gastric carcinoma patients until the second year. Afterward, annual follow-up was performed until the 7th year for early gastric cancer and 10th year for advanced gastric cancer. If necessary, abdominal ultrasonic examination, chest CT scan, whole body bone scan, and PET scan were performed. After that, follow-up studies including EGD were carried out every two years. If patients agreed, annual follow-up studies continued.

### Variables

Stages of RGC and previous gastric cancer were determined according to the 8th edition of the American Joint Commission on Cancer (AJCC). However, sub-stage was not divided due to small sample size. Interval was defined as the time (month) from the previous gastrectomy to RGC surgery. Recurrence site was classified as anastomosis site (gastro-duodenostomy or gastrojejunostomy) and non-anastomosis site. Differentiation was determined by WHO classification and categorized into two groups. The differentiated group included papillary, well or moderately differentiated adenocarcinoma. The undifferentiated group included poorly differentiated adenocarcinoma, mucinous carcinoma, signet ring cell, or cohesive carcinoma. Pathologic report of RGC was reviewed and RGC was classified as intestinal, diffuse, or mixed type according to Lauren classification.

This study was conducted in accordance with the Declaration of Helsinki. It was approved by the Institutional Review Board of Yeungnam University Medical Center, Daegu, Republic of Korea (IRB No. 2018-09-030-001).

### Statistical analysis

All statistical analyses were performed using SPSS version 22.0 (IBM Corporation, Armonk, NY, USA) with significance level set at *p* < 0.05. Continuous variables are expressed as mean ± standard deviation (range) and compared using Student’s *t* test and Cohen’s *d* with 95% confidence interval. Categorical variables are expressed as frequency and compared using chi-square test or Fisher’s exact test and odds ratio (OR) with 95% confidence interval.

## Results

The mean age of 48 RGC-M patients was 64.3 years (range, 39 to 80 years). There were 42 (87.5%) males and 6 (12.5%) females. Twenty-one (43.7%) cases recurred at non-anastomosis site and 27 (56.2%) cases recurred at anastomosis site. The mean length of the previous proximal resection margin (PRM) was 5.9 ± 3.8 cm and the mean interval of 48 RGC-M patients was 105.6 ± 74.7 months (Table 1).

**Table 1:** Baseline characteristics of RGC patients after gastric cancer

Variables	(*n* = 48)
Age (years)	64.3 ± 8.9 (39–80)
Sex	
Male	42 (87.5%)
Female	6 (12.5%)
Interval	
< 5 years	15 (31.3%)
≥ 5, < 10 years	15 (31.3%)
≥ 10 years	18 (37.5%)
Total (months)	105.6 ± 74.7 (14–350)
Reconstruction method	
Billroth I	13 (27.1%)
Billroth II	35 (72.9%)
Previous cancer stage	
Stage I	30 (62.5%)
Stage II	11 (22.9%)
Stage III	7 (14.6%)
Previous PRM (cm)	
< 3	9 (18.8%)
≥ 3	39 (81.3%)
Total	5.9 ± 3.8 (0.5–20)
Previous LI	
Positive	17 (35.4%)
Negative	31 (64.6%)
Previous VI	
Positive	3 (6.3%)
Negative	45 (93.8%)
Recurrence site	
Non-anastomosis	21 (43.8%)
Anastomosis	27 (56.3%)
Differentiation	
Differentiated	21 (43.8%)
Undifferentiated	27 (56.3%)
Lauren classification	
Intestinal	17 (35.4%)
Diffuse	18 (37.5%)
Mixed	13 (27.1%)
RGC stage	
Stage I	24 (50%)
Stage II	12 (25%)
Stage III	12 (25%)

*RGC*, remnant gastric cancer; *PRM*, proximal resection margin; *LI*, lymphatic invasion; *VI*, vascular invasion

Interval of RGC-M according to Lauren classification was 138.5 months for intestinal type, 76.2 months for diffuse type, and 103 months for mixed type, demonstrating statistically significant difference (*p* = 0.044, Cohen’s *d* = − 0.805, − 0.474). Interval of RGC-M in anastomosis site was 132.6 months, which was longer than that (70.8 months) of RGC-M in non-anastomosis site (*p* = 0.002, Cohen’s *d* = 0.953). There was no significant difference in the interval by previous PRM or stage of previous gastric cancer or stage of RGC. There was no significant difference in the interval based on sex or differentiation of RGC either (Table 2).

**Table 2:** Interval of RGC according to variables

Variables	Interval (month)	Effect size (Cohen’ *d*)	95% CI	*p* value
Age (years)				
< 65(*n* = 25)	92.4 ± 67.7 (14–256)	-	-	0.208
≥ 65(*n* = 23)	119.8 ± 80.7 (14–350)	0.369	− 0.202, 0.94	
Sex				
Male (*n* = 42)	109.2 ± 76.1 (14–350)	-	-	0.372
Female (*n* = 6)	79.8 ± 63.7 (15–179)	− 0.393	− 1.252, 0.465	
Previous cancer stage				
Stage I (*n* = 30)	103.0 ± 80.1 (14–350)		-	0.810
Stage II (*n* = 11)	101.7 ± 58.1 (15–225)	− 0.016	− 0.707, 0.673	
Stage III (*n* = 7)	122.8 ± 81.6 (28–256)	0.247	− 0.577, 1.071	
Previous PRM (cm)				
< 3 (*n* = 9)	66.0 ± 77.3	-	-	0.077
≥ 3 (*n* = 39)	114.7 ± 72.0	0.667	− 0.068, 1.404	
Previous LI				0.857
Positive (*n* = 17)	102.9 ± 69.9	-	-	
Negative (*n* = 31)	107.1 ± 78.3	0.054	− 0.536, 0.646	
Previous VI				0.546
Positive (*n* = 3)	80.0 ± 60.25	-	-	
Negative (*n* = 45)	107.3 ± 75.8	0.362	− 0.808, 1.533	
Reconstruction method				
Billroth I (*n* = 13)	67 ± 34.5.5 (14–143)	-	-	0.003
Billroth II (*n* = 35)	119.9 ± 80.7 (14–350)	1.017	0.349, 1.686	
Recurrence site				
Non-anastomosis (*n* = 21)	70.8 ± 53.6 (14–256)	-	-	0.002
Anastomosis (*n* = 27)	132.6 ± 78.3 (14–350)	0.953	0.352, 1.554	
Differentiation				
Differentiated (*n* = 21)	125.6 ± 87.1 (14–350)	-	-	0.102
Undifferentiated (*n* = 27)	90 ± 60.6 (14–221)	− 0.485	− 1.063, 0.09	
Lauren classification				
Intestinal (*n* = 17)	138.5 ± 88.5 (14–350)	-	-	0.044
Diffuse (*n* = 18)	76.2 ± 65.0 (14–206)	− 0.805	− 1.495, − 0.116	
Mixed (*n* = 13)	103.0 ± 50.9 (27–190)	− 0.474	− 1.207, 0.257	
RGC stage				
Stage I (*n* = 24)	109.7 ± 84.2 (14–350)	-	-	0.145
Stage II (*n* = 12)	72.0 ± 47.3 (15–190)	− 0.605	− 1.312, 0.101	
Stage III (*n* = 12)	130.9 ± 69.2 (28–256)	0.266	− 0.429, 0.961	

*RGC*, remnant gastric cancer; *PRM*, proximal resection margin; *LI*, lymphatic invasion; *VI*, vascular invasion; *Calculation of Cohen’s d*, first row category is set to control group and lower row category is set to experimental group

The interval of RGC after Billroth I reconstruction (RGC-B I) was 67 months (5.5 years), which was shorter than that (119 months or 9.9 years) of RGC after Billroth II reconstruction (RGC-B II) (*p* = 0.003, Cohen’s *d* = 1.017) (Table 2). Interval was categorized into lesser than 5 years group, more than 5 years but less than 10 years group, and more than 10 years group for more detailed comparison of interval according to reconstruction method. There were 5 (38.5%) cases of RGC-B I and 10 (28.6%) cases of RGC-B II in lesser than 5 years group, 7 (53.8%) cases of RGC-B I and 8 (22.9%) cases of RGC-B II in more than 5 years to less than 10 years group, and 1 (7.7%) case of RGC-B I and 17 (48.6%) cases of RGC-B II in more than 10 years group. Distribution of interval group of RGC-M according to each reconstruction method demonstrated statistically significant difference (*p* = 0.024, OR = 0.571, 8.500). Also, there was significant difference in recurrence site according to previous reconstruction method (*p* = 0.001, OR = 13.750). Eleven (84.6%) cases of RGC-B I recurred at non-anastomosis site and 2 (15.4%) cases of RGC-B I recurred at anastomosis site. Ten (28.6%) cases of RGC-B II recurred at non-anastomosis site and 25 (71.4%) cases of RGC-B II recurred at anastomosis site. There was no significant difference in the advancement of previous gastric cancer (*p* = 0.151, OR = 5.789, 3.272) or previous PRM (*p* = 0.580, OR = 1.450) between RGC-B I and RGC-B II (Table 3).

**Table 3:** Clinicopathologic features of RGC according to reconstruction method

Variables	RGC-B I (*n* = 13)	RGC-B II (*n* = 35)	*p* value^†^	OR	95% CI for OR	*p* value^‡^
Interval (years)						
< 5	5 (38.5%)	10 (28.6%)	0.024	1	-	-
≥ 5, < 10	7 (53.8%)	8 (22.9%)		0.571	0.130~2.503	0.458
≥ 10	1 (7.7%)	17 (48.6%)		8.500	0.865~83.493	0.066
Recurrence site						
Non-anastomosis	11 (84.6%)	10 (28.6%)	0.001	1	-	-
Anastomosis	2 (15.4%)	25 (71.4%)		13.750	2.574~73.455	0.002
Differentiation						
Differentiated	6 (46.2%)	15 (42.9%)	0.838	1	-	-
Undifferentiated	7 (53.8%)	20 (57.1%)		1.143	0.314~4.109	0.838
Previous cancer stage						
Stage I	11 (84.6%)	19 (54.3%)	0.151	1	-	-
Stage II	1 (7.7%)	10 (28.6%)		5.789	0.651~51.505	0.115
Stage III	1 (7.7%)	6 (17.1%)		3.272	0.369~32.743	0.277
Previous PRM (cm)						
< 3	3 (23.1%)	6 (17.1%)	0.687	1	-	-
≥ 3	10 (76.9%)	29 (82.9%)		1.450	0.304~6.909	0.641
Total	6.4 ± 3.6	5.7 ± 3.9	0.580			
Previous LI						
Positive	3 (23.1%)	14 (40.0%)	0.330	2.222	0.518~9.537	0.283
Negative	10 (76.9%)	21 (60.0%)		1	-	-
Previous VI						
Positive	1 (7.7%)	2 (5.7%)	1.000	0.727	0.060~8.769	0.802
Negative	12 (92.3%)	33 (94.3%)		1	-	-
Lauren classification						
Intestinal	4 (30.8%)	13 (37.1%)	0.904	1	-	-
Diffuse	5 (38.5%)	13 (37.1%)		0.800	0.174~3.669	0.774
Mixed	4 (30.8%)	9 (25.7%)		0.692	0.136~3.518	0.658

*RGC*, remnant gastric cancer; *RGC-B I*, remnant gastric cancer after Billroth I reconstruction; *RGC-B II*, remnant gastric cancer after Billroth II reconstruction; *OR*, odds ratio; *PRM*, proximal resection margin; *LI*, lymphatic invasion; *VI*, vascular invasion; *p value*^*†*^, *p* value for chi-square test; *p value*^*‡*^, *p* value for odds ratio

## Discussion

Although only a few studies have been performed on the incidence of RGC, RGC after gastric cancer (RGC-M) has increased due to increased survival rate of gastric cancer patients according to early detection and improvement in surgical technique and chemotherapy [8]. Many RGC studies have been conducted. However, there is no consensus on characteristics, range of resection, or prognosis of RGC due to problems such as the rareness of RGC and the inconsistency of definitions of RGC. In the current situation where RGC-M is expected to increase, this study is meaningful in that it evaluates characteristics of RGC-M and the occurrence pattern of RGC-M such as recurrence site and interval according to previous reconstruction method.

Most studies about the interval of RGC have reported that the interval of RGC-B is longer than that of RGC-M [9]. It has been proposed that this diversity of interval by initial disease originates from differences in mechanism of carcinogenesis. Environmental factors such as chronic stimulation by bile reflux and mucosa denervation after gastrectomy are responsible for RGC-B occurrence in remnant stomach [10, 11]. These environmental changes can also affect remnant stomach after gastrectomy for malignant disease. In addition to environmental factors, remnant stomach after gastrectomy for malignant disease already has precancerous factors such as atrophic gastritis and intestinal metaplasia or hidden malignancy and metachronous gastric cancer in remnant stomach. A combination of these precancerous factors and environmental factors in remnant stomach after malignant disease contributes to the shorter interval of RGC-M [12]. That is, precancerous factors in remnant stomach are the main causes of diversity of interval between RGC-B and RGC-M.

**Fig. 2:**
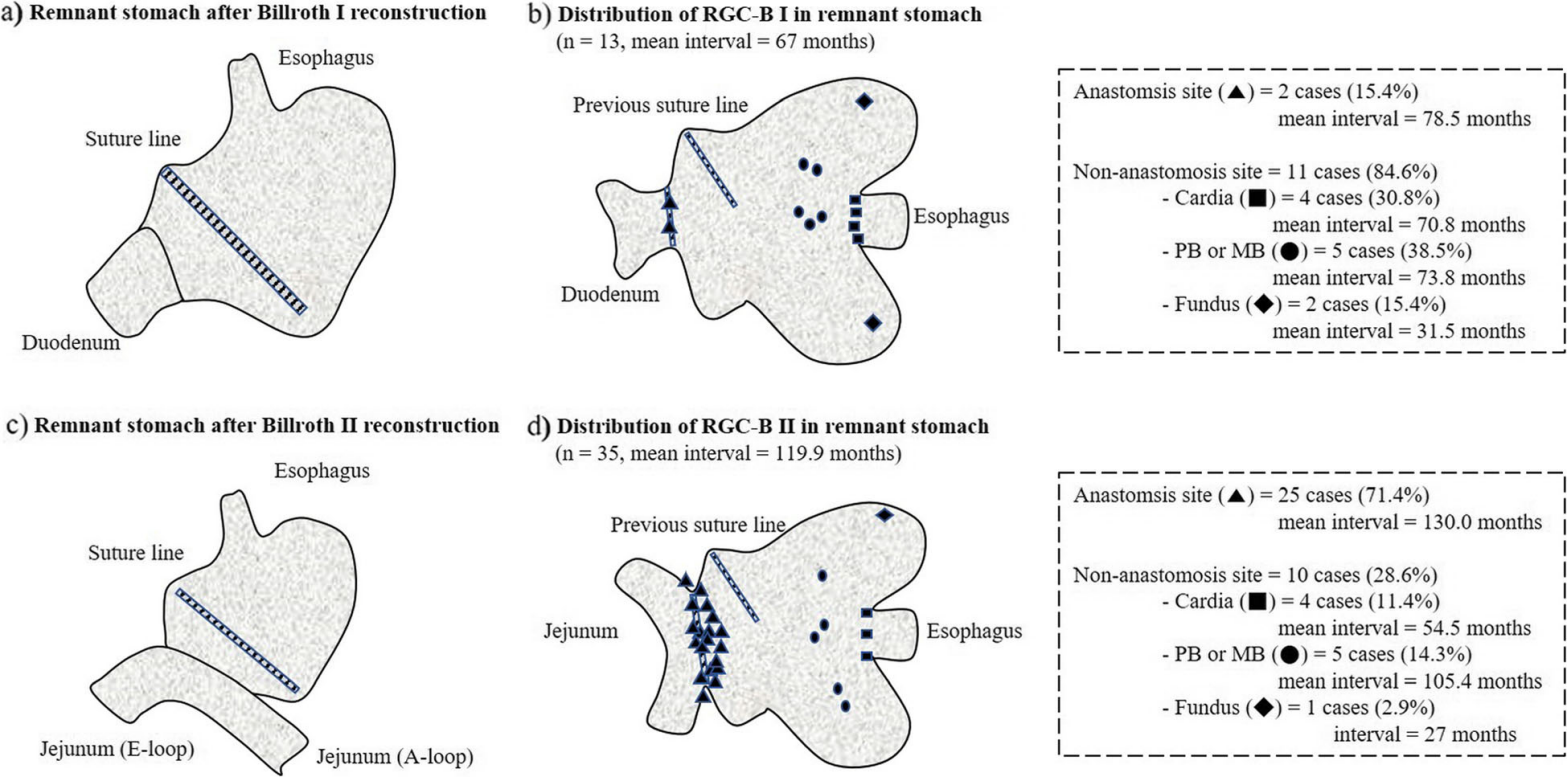
Schematic figure of remnant stomach and the pattern of RGC according to previous reconstruction methods. Anastomosis sites are apart from suture line (transected line) (**a**), (**c**). Most (84.6%) of RGC-B I occurred at non-anastomosis site, especially cardia and PB or MB (**b**). Most (71.4%) of RGC-B II occurred at anastomosis site with long interval (130 months) (**d**). RGC, remnant gastric cancer; PB, proximal body; MB, mid-body; RGC-B I, RGC after Billroth I reconstruction; RGC-B II, RGC after Billroth II reconstruction

**Fig. 3:**
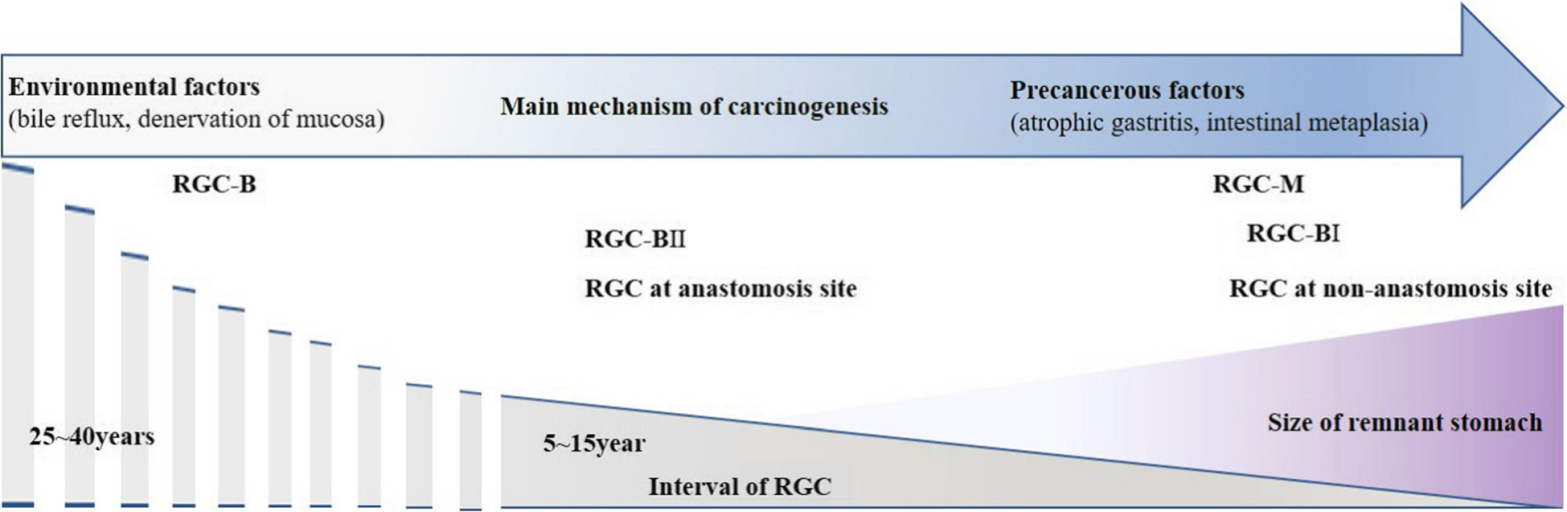
Interval distribution of RGC according to diverse factors. Occurrence pattern and interval of RGC are affected by precancerous and environmental factors. These mechanisms of carcinogenesis are also affected by primary disease, previous reconstruction method, and size of remnant stomach. RGC, remnant gastric cancer; RGC-B, RGC after benign disease; RGC-M, RGC after gastric cancer; RGC-B I, RGC after Billroth I reconstruction; RGC-B II, RGC after Billroth II reconstruction

The interval of RGC-B I is also significantly shorter than that of RGC-B II in most reported studies [13, 14]. In our study, although RGC-B was excluded, the mean interval of RGC-B I was 67 months (5.5 years), which was shorter than that (119 months or 9.9 years) of RGC-B II (*p* = 0.003, Cohen’s *d* = 1.017) (Table 2). Both the interval of RGC-M and the recurrence site of RGC-M were related to previous reconstruction methods (Table 3). We found that RGC-B I showed frequent recurrences around the non-anastomotic site (11 out of 13, 84.6%) while RGC-B II showed a tendency to often recur at the anastomotic site (25 out of 35, 71.4%) (*p* = 0.001, OR = 13.750), consistent with results of other studies [9, 14] (Fig. 3). Considering the tendency of RGC to occur at different locations according to each reconstruction method, mechanisms of carcinogenesis in remnant stomach are different from those at anastomosis site after Billroth II and those at non-anastomosis site after Billroth I. Usually, the size of remnant stomach after Billroth I is larger than that after Billroth II to make for tension-free gastro-duodenostomy. Increase in the size of remnant stomach can raise the possibility of remnant stomach to have precancerous lesion with genetic vulnerability to RGC. Therefore, precancerous factor might be the main mechanism contributing to the carcinogenesis of RGC-B I with short interval at non-anastomosis site. In remnant stomach after Billroth II, it is considered that lower possibility of precancerous lesion and frequent bile reflux through gastrojejunostomy are mechanisms of carcinogenesis. Thus, the interval of RGC-B II at anastomosis site is longer than the interval of RGC-B I at non-anastomosis site due to weaker precancerous factor and stronger environmental factor than that after Billroth I reconstruction (Fig. 2).

As previously mentioned in the method, the final decision of the reconstruction method was done after visualization of the cancer lesion. Oncologic safety and tension-free anastomosis were decisive factors. To secure pathologically negative resection margin, 2- to 3-cm margin length is usually enough for early gastric cancer and over 5-cm margin length is required for advanced gastric cancer. If the location of the cancer lesion is appropriate for tension-free gastro-duodenostomy with enough safety margin, Billroth I is preferred as a reconstruction method. If not, then Billroth II is preferred as a reconstruction method. Although previous reconstruction method was different, there was no significant difference in the length of previous PRM between RGC-B I and RGC-B II (*p* = 0.580) (Table 3). If the resection margin is pathologically negative, the length of the resection margin does not affect the prognosis and local recurrence of gastric cancer patients [15, 16]. In this study, there was no significant difference in the interval of RGC by the length of previous PRM (*p* = 0.077, Cohen’s *d* = 0.667) (Table 2). Thus, it is thought that the interval of RGC occurrence is not influenced by the length of pathologically negative PRM.

Surprisingly, there was no correlation between stages of the previous gastric cancer and the interval of RGC-M (*p* = 0.810, Cohen’s *d* = − 0.016, 0.247) in this study (Table 2). In other words, the invasion depth of the previous gastric cancer or the degree of LN metastasis did not significantly affect the interval of RGC-M. Thus, we presume that the interval of RGC in remnant stomach is not influenced by the stage of previous gastric cancer, but by mechanisms of carcinogenesis such as environmental factors and precancerous factors. However, more data and genetic mutational studies on carcinogenesis of RGC-M are needed to prove this hypothesis. Also, more active surveillance will be needed after gastrectomy in patients with family history of gastric cancer or genetic mutation.

Many studies have been conducted on clinicopathologic features of RGC, reporting that RGC-M has several distinguishing features compared with primary gastric cancer. In RGC-M patients, there could be changes in lymphatic flow around remnant stomach by previous LN dissection. Such lymphatic flow changes might lead to alteration of LN metastasis pattern [17]. Also, the number of retrieved LN during RGC surgery is smaller than the number of retrieved LN during D2 dissection for primary gastric cancer. Thus, N category of TNM system for primary gastric cancer can not reflect the prognosis or the degree of LN metastasis in RGC-M patients. Although several studies have reported modified staging system using LN ratio, the utility of these alternative systems is not superior to TNM system for primary gastric cancer [18, 19]. Considering these disease distinctions of RGC, another staging system and surgical guideline for LN dissection or extent of gastric resection are needed. Guideline of surgery and staging system for RGC are essential for correct analysis and comparison of data. Further well-designed large-scale studies on genetic mutation of RGC patients are possible on the basis of these foundations.

Open total gastrectomy was performed in this study. However, laparoscopic approach has been performed as a surgical option for RGC in other studies. Laparoscopic total gastrectomy for RGC has lesser blood loss and fewer post-op complications than the open approach. The 5-year survival rate of RGC patients with laparoscopic total gastrectomy was similar to that of patients with open total gastrectomy [20, 21]. However, there are only a few studies on the laparoscopic surgery of RGC. In addition, their sample sizes were small. Thus, further studies with large sample size are required.

Some researchers have found that the prognosis of early RGC is not worse than that of early primary gastric cancer, although the prognosis of advanced RGC is worse than that of advanced primary gastric cancer [22]. To obtain good prognosis of RGC, it is important not only to create new staging system and surgical guideline for RGC, but also to perform regular surveillance after gastrectomy for early diagnosis and treatment of RGC.

In the author’s institution, surveillance after gastrectomy has been carried out annually until the 7th year for early gastric cancer and the 10th year for advanced gastric cancer. Thereafter, surveillance study was done every two years. Considering the facts that more than one-third of RGC occurred more than 10 years after the initial gastrectomy and there was no correlation between the interval of RGC-M and stage of previous gastric cancer, regular surveillance should be performed annually after the 10th year of gastrectomy for early diagnosis and treatment of RGC. Considering the distribution and interval of RGC in remnant stomach based on previous reconstruction methods (Fig. 3), we should observe carefully cardia and lesser curvature from early period of surveillance in patients with Billroth I reconstruction. Additional focus on anastomosis site is needed from the late period of surveillance in both patients with Billroth I reconstruction and patients with Billroth II reconstruction.

This study has several limitations. First, Roux-en-Y reconstruction, a commonly used reconstruction method, was not included in this study. In most studies comparing Roux-en-Y with Billroth reconstruction methods, remnant stomach after Roux-en-Y reconstruction showed lower incidence of reflux gastritis than Billroth reconstruction methods [23, 24]. Thus, it is thought that environmental factor after Roux-en-Y reconstruction is weaker than that after Billroth reconstruction. In addition, the size of remnant stomach after Roux-en-Y reconstruction is smaller than that after Billroth I reconstruction. Hence, precancerous factor after Roux-en-Y reconstruction seems to be weaker than that after Billroth I reconstruction. These different mechanisms of Roux-en-Y reconstruction with discrepancies in environmental and precancerous factors might be helpful in comprehending mechanisms of carcinogenesis in remnant stomach. Second, this study was a retrospective small sample sized study with selection bias. Unfortunately, we could not match clinicopathologic features affecting carcinogenesis in both reconstruction method groups due to small sample size. Hence, this study has limitation in the reliability of results. Based on results of this study, we have started a large-scale multi-center study with five tertiary referral university hospitals in Daegu, Korea (Daegu Gastric Cancer Study Group) from December 2018. Third, detailed analysis of splenectomy and metastasis pattern of LN, especially mesenteric LN and LN 10, was not performed in this study. Thus, it is difficult to conclude whether there are beneficial effects of survival following splenectomy, dissection of mesenteric LN, or LN 10 stations. Fourth, we found that the interval and recurrence site of RGC were affected by multifarious factors involving precancerous and environmental factors. However, we could not perform genetic studies to prove mechanisms of carcinogenesis in remnant stomach in this study. Therefore, further large-scale, well-designed study with genetic examinations is needed to understand the precise mechanism of carcinogenesis in remnant stomach.

## Conclusion

RGC after Billroth I reconstruction tends to arise earlier at non-anastomosis site than RGC after Billroth II. Therefore, we should examine non-anastomosis site carefully from the beginning of surveillance after gastric cancer surgery with Billroth I reconstruction for better outcome.

## Data Availability

The anonymized data used and/or analyzed during the current study are available from the corresponding author on reasonable request.

## Abbreviations

RGC: Remnant gastric cancer
YUMC: Yeungnam University Medical Center
RGC-B: Remnant gastric cancer after benign disease
RGC-M: Remnant gastric cancer after gastric cancer
EGD: Esophagogastroduodenoscopy
OR: Odds ratio
PRM: Proximal resection margin
RGC-B I: Remnant gastric cancer after Billroth I reconstruction
RGC-B II: Remnant gastric cancer after Billroth II reconstruction
PB: Proximal body
MB: Mid-body
